# Machine Learning Techniques Disclose the Combined Effect of Fermentation Conditions on Yeast Mixed-Culture Dynamics and Wine Quality

**DOI:** 10.3390/microorganisms10010107

**Published:** 2022-01-05

**Authors:** Catarina Barbosa, Elsa Ramalhosa, Isabel Vasconcelos, Marco Reis, Ana Mendes-Ferreira

**Affiliations:** 1 CoLAB VINES&WINES—National Collaborative Laboratory for the Portuguese Wine Sector, Associação para o Desenvolvimento da Viticultura Duriense (ADVID), Edifício Centro de Excelência da Vinha e do Vinho, Régia Douro Park, 5000-033 Vila Real, Portugal; crbarbosa@utad.pt; 2 BioISI—UTAD, Biosystems & Integrative Sciences Institute, WM&B—Laboratory of Wine Microbiology & Biotechnology, Department of Biology and Environment, Universidade de Trás-os-Montes e Alto Douro, 5000-801 Vila Real, Portugal; 3 Mountain Research Centre (CIMO), ESA—Polytechnic Institute of Bragança, Campus de Sta Apolónia, 5300-253 Bragança, Portugal; elsa@ipb.pt; 4 CBQF/Centro de Biotecnologia e Química Fina, Escola Superior de Biotecnologia, Universidade Católica Portuguesa, 4169-005 Porto, Portugal; ivasconcelos@porto.ucp.pt; 5 Department of Chemical Engineering, University of Coimbra, CIEPQPF, Rua Sílvio Lima, Pólo II—Pinhal de Marrocos, 3030-790 Coimbra, Portugal; marco@eq.uc.pt; 6 CITAB—Centre for the Research and Technology of Agro-Environmental and Biological Sciences, Universidade de Trás-os-Montes e Alto Douro, 5000-801 Vila Real, Portugal

**Keywords:** supervised and unsupervised machine learning, non-*Saccharomyces* yeasts, nitrogen, sugar, temperature, aroma production, central composite design

## Abstract

The use of yeast starter cultures consisting of a blend of *Saccharomyces cerevisiae* and non-*Saccharomyces* yeasts has increased in recent years as a mean to address consumers’ demands for diversified wines. However, this strategy is currently limited by the lack of a comprehensive knowledge regarding the factors that determine the balance between the yeast-yeast interactions and their responses triggered in complex environments. Our previous studies demonstrated that the strain *Hanseniaspora guilliermondii* UTAD222 has potential to be used as an adjunct of *S. cerevisiae* in the wine industry due to its positive impact on the fruity and floral character of wines. To rationalize the use of this yeast consortium, this study aims to understand the influence of production factors such as sugar and nitrogen levels, fermentation temperature, and the level of co-inoculation of *H. guilliermondii* UTAD222 in shaping fermentation and wine composition. For that purpose, a Central Composite experimental Design was applied to investigate the combined effects of the four factors on fermentation parameters and metabolites produced. The patterns of variation of the response variables were analyzed using machine learning methods, to describe their clustered behavior and model the evolution of each cluster depending on the experimental conditions. The innovative data analysis methodology adopted goes beyond the traditional univariate approach, being able to incorporate the modularity, heterogeneity, and hierarchy inherent to metabolic systems. In this line, this study provides preliminary data and insights, enabling the development of innovative strategies to increase the aromatic and fermentative potential of *H. guilliermondii* UTAD222 by modulating temperature and the availability of nitrogen and/or sugars in the medium. Furthermore, the strategy followed gathered knowledge to guide the rational development of mixed blends that can be used to obtain a particular wine style, as a function of fermentation conditions.

## 1. Introduction

The use of starter cultures of active dry yeast of *Saccharomyces cerevisiae* is a common practice in wine-producing regions, providing reliable and reproducible fermentations that circumvent vintage variability. However, there is a consumer perception that this practice has led to a loss of wine flavor complexity and stylistic distinction. In this context, several studies have assessed the contribution of natural non-*Saccharomyces* yeasts to the final quality of wines [1,2,3,4,5], showing that these yeasts possess several interesting metabolic properties and enzymatic activities that are less marked or even absent in *S. cerevisiae* and that significantly contribute to the aromatic profile of wines [5,6,7,8]. For this reason, there has been increasing effort devoted to the development of new starter cultures, based on selected non-*Saccharomyces* yeasts, to be used as adjuncts of *S. cerevisiae* in grape juice fermentations to enhance wine’s flavor and character [9,10,11]. Nevertheless, the success of using these mixed starter cultures will be greatly dependent on grape juice composition and fermentation conditions, as they determine individual yeast growth, interactions between the different strains involved and ultimately their contribution to the final composition of the wines. The effect of nitrogen availability on the growth and fermentation kinetics of wine yeast, together with its impact on fermentative aroma production, has been comprehensively studied in recent decades in *S. cerevisiae* [12,13,14,15]. Many volatile and non-volatile compounds produced by *S. cerevisiae* that constitute wine’s bouquet, such as higher alcohols and fatty acids and their associated esters, have been found to be highly dependent not only on the amount but also on the nature of nitrogen available [16,17,18]. More recently, it has been shown that non-*Saccharomyces* yeasts differ in their demands and preferences for nitrogen sources available in grape juice, which has an impact on their individual contribution to secondary aroma production in wines [3,19]. In mixed-culture fermentations, it has been demonstrated that co-inoculation of non-*Saccharomyces* strains may lead to faster nitrogen consumption. This can hamper the growth and fermentative ability of *S. cerevisiae* strains [17,20,21,22] and modify the production of fermentative aroma compounds by *S.* *cerevisiae* [17,23,24,25].

Another critical variable is the concentration of fermentable sugars in grape juice, ranging from 150 to 300 g/L [26]. Currently, due to global warming, we are witnessing a significant increase in the initial concentrations of sugars in grape musts, glucose, and fructose, which selectively influence the species and/or strains of yeasts that are better adapted to grow under such conditions [27]. Additionally, it is expected that initial sugar fermentation will significantly impact the production of several metabolites with relevance for the wine complexity and quality. Indeed, fusel alcohols and esters [28], acetic acid [29], and glycerol [30] are mainly produced in parallel to ethanol by *S. cerevisiae* through fermentation of sugars. Besides the direct effect of sugar concentration on ethanol levels, which will determine the persistence of non-*Saccharomyces* yeasts during mixed culture fermentations [31,32], the many physiological studies on the contribution of these yeasts to the aromatic profile of wines have shown that non-*Saccharomyces* yeasts display metabolic diversity relative to *S. cerevisiae* [11]. Recent reports at the genomic level of different non-conventional wine-related yeasts [33,34] have indicated potential mechanisms such as alterations in the regulation of gene expression, absence of homologous genes, gene duplications and/or modification of enzymatic activities, which may underly the differences observed.

The dynamics of yeast populations during alcoholic fermentation is also affected by the temperature of vinification. It has been established that temperatures above 15 °C provide *S. cerevisiae* with an advantage that allows it to outcompete non-*Saccharomyces* yeasts, while lowering the temperature of fermentation enhances the growth, survival, and thus the competitive ability of non-*Saccharomyces* yeasts, as their susceptibility to ethanol is attenuated in these condition [35,36,37]. Fermentation temperature is also known to affect the fermentation rate and the *S. cerevisiae* metabolism, influencing the overall aroma profile of a wine [38]. A study conducted under two different temperatures (13 and 28 °C) [39] showed that, at the lowest temperature, *S. cerevisiae* enhanced the production of acetate esters, fatty acids, and their corresponding ethyl esters, and decreased volatile acidity and fusel alcohol concentrations. Despite the importance of fermentation temperature on non-*Saccharomyces* yeast survival and persistence during fermentation, there is still a lack of knowledge of the impact of fermentation temperature on the metabolism of these yeasts.

The overall effects of non-*Saccharomyces* strains in mixed-culture fermentation are highly dependent on the inoculation strategy as it strongly influences the dominance and persistence of yeast species during fermentation and thus their contribution to wine traits. Two main inoculation approaches of non-*Saccharomyces* and *S. cerevisiae* strains have generally been studied: (i) simultaneous, where both yeast species are inoculated at the same time before fermentation, and (ii) sequential inoculation, where the non-*Saccharomyces* yeast is initially inoculated followed by the inoculation of *S. cerevisiae*. Although simultaneous co-inoculation can offer the advantage of avoiding a second inoculation stage, which increases the operation time and operational steps in the winery, yeast manufacturers presently recommend the sequential inoculation protocol to apply the commercial formulation already available on the market. This option is based on the evidence that this strategy promotes the persistency and individual contribution of the non-*Saccharomyces* yeast to the modulation of the sensory profile, and/or to reduce the final alcohol content in wine (reviewed in [40]).

The effects of fermentation conditions on yeast performance and wine quality have traditionally been studied altering one variable at a time. This approach is inefficient, because it cannot discern the presence of interactions among the experimental factors, making it difficult or even impossible to provide an accurate assessment of their impact on wine fermentations. Based on the previous knowledge, this caveat is even more evident when mixed-culture fermentations are used. In a previous work, our group assessed the potential of a selected a *Hanseniaspora guilliermondii* strain to be used as a starter culture in mixed-culture fermentations [17]. The experiments conducted in a natural grape juice showed that, although the simultaneously co-inoculation of *H. guilliermondii* UTAD222 negatively affected *S. cerevisiae* growth and fermentation rate, its presence significantly altered the panoply of aroma compounds found at the end of the fermentation [17]. Intending to increase knowledge of this yeast consortium and to evaluate the suitability of its application in different winemaking conditions, in the present study we sought to analyze the influence of *H. guilliermondii* UTAD222 strain and its interaction with other production factors (nitrogen, sugar and temperature) on fermentation kinetics and on the production of sensory relevant metabolites. The experimental plan was carried out using a Central Composite Design methodology to optimize the number of experiments required to evaluate the effects of these four factors and their putative interactions. Given that the winemaking environment is a complex biological system, it is expected to present the abstract characteristics of modularity, hierarchy, and functional specificity [41,42,43,44]. These systems are not fully characterized by single responses or parameters, but by multiple dimensions reflecting different aspects of the biological mechanisms. Therefore, data analysis methods should conform to such conditions and be able to extract the relevant information conveyed in data [44]. In this setting, responses are more appropriately analyzed multivariately than univariately, and, furthermore, adequate methodologies should be developed to flexibly infer the existing modules and their relationship. These aspects were considered in this work, where response modules were first identified and summarized using unsupervised machine learning techniques. Then, the collective dependence of the responses in each module upon the experimental factors was determined through supervised methodologies, leading to predictive models for the behavior of each functional cluster. With the developed predictive models, it is not only possible to infer which factors are critical to each module and how they affect them, but also to predict the underlying latent variable response and, upon reconstruction, the values of all correlated variables in each cluster.

The proposed advanced data analysis methodology used herein will pave the way for the rational use of mixed cultures while providing practical guidelines for winemakers to adjust mixed starter cultures as a function of winemaking conditions towards the controlled production of new, diversified, and tailor made wines.

## 2. Materials and Methods

### 2.1. Yeasts Strains and Maintenance Conditions

The strain *H. guilliermondii* UTAD222, previously isolated in our laboratory from a fermenting grape juice from Douro Region [45], was selected for this study for its interesting oenological traits such as high ethanol tolerance and low potential for hydrogen sulfide production. *S. cerevisiae* UCD522 was supplied by the Enology Culture Collection, Department of Viticulture and Enology, University of California, Davis, CA, USA. Pure cultures were routinely maintained at 4 °C on Yeast Peptone Dextrose (YPD) slants, containing glucose (20 g/L), peptone (10 g/L), yeast extract (5 g/L) and agar (20 g/L), and the stocks were stored at −80 °C with glycerol (40% *v*/*v*).

### 2.2. Fermentation Media

In this study, the chemically defined Grape juice Medium (GJM) used was similar in composition to typical grape juice previously described elsewhere [46], with some modifications. Three different levels of initial yeast assimilable nitrogen (YAN) and sugar content were used in the experimental design (Table 1). YAN was supplied as a mixture of amino acids and ammonium in a proportion of 60:40%, at final concentrations of 100, 300, or 500 mg/L. Ammonium was supplied as di-ammonium phosphate (DAP). The carbon and energy source was composed of an equimolar mixture of glucose and fructose (1:1) at final concentrations of 150, 225, or 300 g/L.

### 2.3. Fermentation Trials

For all experiments, starter cultures were prepared by pre-growing the yeasts overnight in 100 mL shake flasks containing 70 mL of YPD with pH adjusted to 3.5 with KOH 10 M. The flasks were incubated overnight at 25 °C in an orbital shaker set at 150 rpm. These starter cultures of *S. cerevisiae* and *H. guilliermondii* were used for the inoculation of GJM at the cell count considered for each experimental run. Inoculation strategy consisted of an unchanging cell count of 5 × 10^5^ CFU/mL for *S. cerevisiae* UCD522 and changing cell counts of *H. guilliermondii* UTAD222: no inoculation (single culture of *S. cerevisiae*), 5 × 10^5^, and 1 × 10^6^ CFU/mL. The fermentations were conducted in 250 mL flasks filled to 2/3 of their volume fitted with a side-arm port sealed with a rubber septum to allow anaerobic sampling. To avoid the accumulation of medium in the system, a stylet was inserted in the needle holder. The flasks were incubated at the appropriate temperatures (10–30 °C) in an orbital shaker set at 120 rpm. Fermentations were monitored daily by weighing the fermentation flasks on a laboratory scale. Growth parameters and analytical determinations were determined after aseptic sampling using a syringe-type system. The end of fermentation, corresponding to the time necessary to reach dryness (R100), was determined assuming that weight loss corresponds to CO_2_ evolution, which is proportional to sugar consumption. To this end, fermentations were allowed to proceed until no further weight loss was observed. Maximum Fermentation Rate (MFR) was determined from the slope of the linear dependence of the steepest decline in weight (g) at the corresponding time points (h). After fermentation, the wines were centrifuged (10 min at 5500 rpm, Sigma 3-18K refrigerated Centrifuge, 37520 Osterode am Harz, Germany) to remove yeast cells and were kept at −20 °C until the analytical determinations were performed. The yeast growth was followed by counting the colony-forming units (CFUs) in YPD agar, WL nutrient agar medium (Wallerstein’s Laboratory), and/or Lysine agar (Oxoid) plates.

### 2.4. Analysis of Fermentation Metabolites by Liquid Chromatography

The levels of glucose, fructose, ethanol, glycerol and acetic acid were determined using a high-performance liquid chromatography (HPLC Flexar, PerkinElmer, Shelton, CT, USA) system equipped with the ion exclusion cation exchange column Aminex HPX-87H (Bio-Rad Laboratories, Hercules, CA, USA) and refractive index and UV/VIS detectors. The column was eluted using sulfuric acid (0.005 N) at 60 °C and a 0.6 mL/min flow rate. The samples were filtered through a membrane (Millipore, 0.22 μm pore size) before injecting 6 μL. The components were identified through their relative retention times compared with the respective standards, using the Chromera Software version 4.1.0; PerkinElmer, Shelton, CT, USA, 2013.

### 2.5. Analysis of Volatile Compounds by Gas Chromatography/Mass Spectrometry

At the end of alcoholic fermentation, samples were taken from different fermented media to be screened for aroma compounds production. Aliphatic higher alcohols (1-propanol, 2-methyl-1-butanol and 3-methyl-1-butanol) and ethyl acetate were analyzed as described by Moreira et al. [47] using a Hewlett Packard 5890 gas chromatograph equipped with a flame ionization detector and connected to a H.P. 3396 Integrator. A 50 μL volume of 4-methyl-2-pentanol at 10 g/L was added to 5 mL of wine as internal standard. The sample (1 μL) was injected (split, 1:60) into a CP-WAX 57 CB column (Chrompack) of 50 m × 0.25 mm and 0.2 μm phase thickness. The temperature program followed was: 40 °C (5 min) to 80 °C (0 min) at 3 °C/min and from 80 °C to 200 °C (4 min) at 15 °C/min. Injector and detector temperatures were set at 220 °C. The carrier gas was H^2^ at 1–2 mL/min.

The determination of 2-phenylethanol, acetates of higher alcohols (isoamyl acetate, 2 phenylethyl acetate), and ethyl esters of fatty acids (ethyl butyrate, ethyl hexanoate, ethyl octanoate, ethyl decanoate and ethyl dodecanoate) was performed in a Hewlett Packard 5890 gas chromatograph, equipped with a flame ionization detector. For that purpose, 50 mL of wine, with 4-decanol at 1.5 mg/L as internal standard, was extracted successively with 4, 2 and 2 mL of etherhexane (1:1 *v*/*v*) for 5 min. The organic phase (1 μL) was injected (splitless) into a BP21 (SGE) column of 50 m × 0.22 mm and a phase thickness of 0.25 μm. The temperature program was 40 °C (1 min) to 220 °C (15 min) at 2 °C/min. Injector and detector temperatures were set at 220 °C. The carrier gas used was H^2^ at 1–2 mL/min.

### 2.6. Experimental Design

A single block central composite design (CCD) methodology with an α value equal to 1 (α is the distance in coded units from the center of the design space to each star point of the CCD design) was conducted to investigate the influence of the four experimental factors on the metabolic profile resulting from the fermentation process. The experimental factors (input variables) were initial sugar content, initial YAN concentration, temperature of fermentation and inoculum level of the non-*Saccharomyces* strain *H. guilliermondii* UTAD222. The responses (output variables) comprised: R100, MFR, acetic acid, glycerol, ethanol as well as several aroma compounds produced, enumerated in Section 2.5. Analysis of Volatile Compounds by Gas Chromatography/Mass Spectrometry. These responses, and their determination, were described in the preceding subsections. Being a response surface type of experimental design, three levels are considered for the experimental factors, usually referring to coded units as −1, 0, or +1, where −1 corresponds to the low level of each factor, 1 to the high level, and 0 to the mid-level. The correspondence between the coded and original levels of the factors is presented in Table 1. The execution of the trials corresponding to the CCD led to thirty-one experiments (Table 2), including seven replicates at the central point (experimental runs, 1, 5, 14, 19, 20, 28 and 30). To minimize the introduction of systematic biases, all experiments were conducted randomly.

### 2.7. Data Analysis Workflow

The data obtained were analyzed using a latent variable framework in order to explore the underlying structure of the response space and model its dependence from the factors considered in the CCD performed. The analysis was conceived in such a way to unravel the modular structure of the responses analyzed and their collective dependence upon the experimental factors. The analysis workflow consisted of three main stages:

Stage 1. Exploratory data analysis (EDA). The results obtained were explored with several univariate and multivariate visualization tools to analyze the main variation and association patterns and pinpoint outliers and any potential faulty measurements that need to be considered in detail. The associations of interest are related not only to the mapping of inputs to outputs, but also the relationships between all 18 outputs.

Stage 2. Unsupervised machine learning. In this stage, the EDA activity was further deepened by applying unsupervised machine learning technique to the entire experimental data set. The multivariate structure of the responses was explored using latent variable methods, such as Principal Component Analysis (PCA), and the associated diagnosis tools (scree plot, scores plot, loadings plots, residual analysis, etc.) [48,49].

Then, natural clusters formed by the set of 18 responses were identified using agglomerative hierarchical clustering (AHC). The distance metric was the correlation distance, as the aim was to find blocks (i.e., clusters or groups) of functionally related fermentation metabolites/conditions. Finally, each identified block was summarized by the linear combination that most explains its correlation structure. These linear combinations were obtained by resorting to PCA, which was applied at the cluster level, i.e., to the clusters obtained with AHC. As clusters are formed by highly associated variables, it was expected (from the properties of PCA; see references above) that the first principal component for the cluster-specific PCA models would explain most of the total variability in the cluster. Therefore, in this work, we used the first principal component for the PCA model (PC1) in each cluster as a surrogate of all the variables inside the cluster. The values of PC1, also known as the PC1 scores, summarize the main variation pattern in each cluster and can be used to reconstruct the original values of the variables; this can be done by simply taking the outer product of the PC1 scores and PC1 loading vector—an operation called reconstruction.

Stage 3. Supervised machine learning. In this final stage, models were developed for each cluster to explain the variability of the cluster’s first principal component, the terms of the experimental factors, and their mutual interactions. As such, we implemented a forward stepwise variable selection strategy in order to the set of input variables (experimental factors and their higher-order terms) to be incorporated into the regression model. In brief terms, the stepwise selection strategy consisted of successively selecting and incorporating factors (and other higher-order terms), as long as they improved the capability to explain the behavior of PC1 to an extent that was statistically significant (evaluated with a partial F-test; reference significance level of 0.05). On the other hand, terms that have already been selected for incorporation into the model in earlier stages may also be discarded later if their contribution stops being significant. The procedure stops when there are no variables whose inclusion or removal from the model will lead to a change in the model’s explanatory power that is statistically significant. It is important to highlight that the primary goal of the models obtained in this way is to identify the factors and interactions that are most significant for explaining the variability of each cluster. This aspect is more important than the accuracy of their prediction, which may require a larger amount of experience to improve.

## 3. Results and Discussion

The main goal of this work was to understand the influence of production factors in shaping wine fermentation and final composition through the evaluation of the combined effects of initial sugar and YAN concentrations, fermentation temperature and co-inoculation with *H. guilliermondii* UTAD222 on fermentation activity and production of volatile and non-volatile compounds using a CCD methodology. This encompassed the identification of the significant terms involving the input variables on a linear regression model for predicting the responses. However, the set of responses to be analyzed amounts to 18 chemical compounds and fermentation parameters, and they are neither independent nor fully correlated. Therefore, classical univariate regression methods, as well as the more advanced multivariate algorithms, are limited in the analysis they can provide for the collective behavior of the responses. The responses are expected to exhibit patterns of modularity and functional affinity, which requires a different analysis workflow than that usually followed in the literature. In this section, we present the main outcomes of the three-stage analytical workflow put forward for handling complex systems, such as the one considered in this work involving mixed-culture fermentations.

### 3.1. Exploratory Data Analysis

Thirty-one experiments were conducted (Table 2) to evaluate the collective effect of initial YAN and sugar concentrations, temperature, and inoculation level of *H. guilliermondii* UTAD222 on the fermentation dynamics and metabolites produced (Table S1). To provide an overview of the impact of process conditions on responses, the results obtained were first analyzed visually.

**Figure 1:**
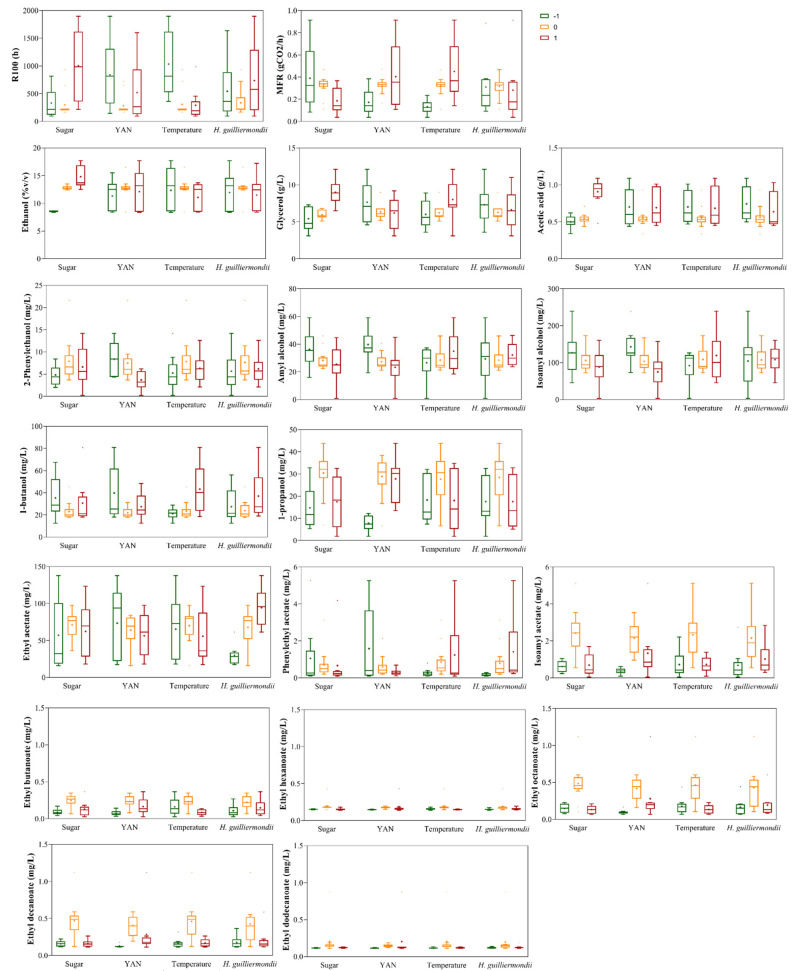
Boxplots exploring the relationships between the 18 responses and each level (−1, 0 and +1) of the factors tested: Sugar, YAN, Temperature, and *H. guilliermondii* UTAD222 inoculum. −1: 150 g/L Sugars, 100 mg/L YAN, 10 °C, 0 cells/mL *H. guilliermondii*; 0: 225 g/L Sugars, 300 mg/L YAN, 20 °C, 5 × 10^5^ cells/mL *H. guilliermondii*; 1: 300 g/L Sugars, 500 mg/L YAN, 30 °C, 1 × 10^6^ cells/mL *H. guilliermondii*. Boxplots indicate, for each response, the values by each factor and corresponding level, showing the mean values (plus symbol) and upper and lower quartiles, with vertical bars showing the minimum and maximum value, respectively.

To visualize the individual relevance of each factor and direction of its impact, in Figure 1, boxplots are presented indicating, for each of 18 responses and considering all 31 fermentation conditions, the distribution values for the three levels of each factor. As illustrated, there were wide variations in the responses as a function of the levels of the four factors tested, clearly underlining their individual impact on fermentation outcome. Fermentation length (R100) and maximum fermentation rate (MFR), considered in this work as summary quantities to study the influence of the experimental factors on the fermentation kinetics, were impacted by all factors. The boxplots for R100 and MFR showed different mean values but overlapping distributions for all fermentation factors studied. Nevertheless, initial sugar concentration and temperature were the factors with the strongest effects on fermentation kinetics, demonstrated by the great disparity in the mean values, and narrow overlapping distributions in the boxplots of their levels. Accordingly, R100 ranged from 96 to 1896 h, with the highest initial concentrations of sugar and lowest temperature being associated with longer fermentations, where yeast cells were unable to consume all sugars (Table S1). Assessing the impact of YAN or inoculum *of H. guilliermondii* on fermentation kinetics, it was possible to verify that variations in their levels resulted in higher differences, mostly with respect to R100 values, suggesting that the length of fermentation may be governed by an interaction of all the conditions, particularly of the initial nitrogen content and the presence of the non-*Saccharomyces* yeast strain. Similarly, the production of the primary products of alcoholic fermentation, ethanol, glycerol, and acetic acid also showed a large impact of sugar concentration, demonstrated in the boxplots with huge variation on the distribution and mean values, contrarily to the overlapping distributions of the boxplots corresponding to the levels of YAN, temperature and *H. guilliermondii* inoculum.

The production of amyl and isoamyl alcohols, along with 2-phenylethanol and the corresponding acetate, was mostly affected by the YAN levels, reaching higher values at the lowest level of initial nitrogen (Figure 1, Table S1). The effect of *H. guilliermondii* UTAD222 inoculation was not so clear, except for 2-phenylethyl acetate, content of which was clearly increased in co-inoculated fermentations (Table S1), as depicted in the boxplots, where the highest mean value was observed for level 1 of the *H. guilliermondii* inoculum, albeit with a huge variation in the distribution. Likewise, the individual impact of the factors on ethyl acetate concentration was more evident for inoculum level (Figure 1, Table S1), being highly variable in the distribution, but with similar mean values at different temperature, sugar, and YAN levels. The production of isoamyl acetate and ethyl esters was shown to be positively regulated by all factors at moderate levels (level 0), where higher concentrations were detected (Figure 1, Table S1). The individual impact of the factors was more obvious on isoamyl acetate concentration, as demonstrated by the greater differences between the mean values obtained for the three levels of each factor studied, especially in YAN and inoculum of *H. guilliermondii*.

Overall, the large variation observed in the distribution of concentrations of the metabolites as a function of levels of each factor tested may reflect that the individual impact could not be measured, suggesting an interaction between the fermentation conditions in the modulation of these compounds. In this way, this first exploration of the responses to the different fermentation conditions demonstrated combined correlations/interactions, supporting the inevitability of a more detailed analysis of the functionally related response variables.

**Figure 2:**
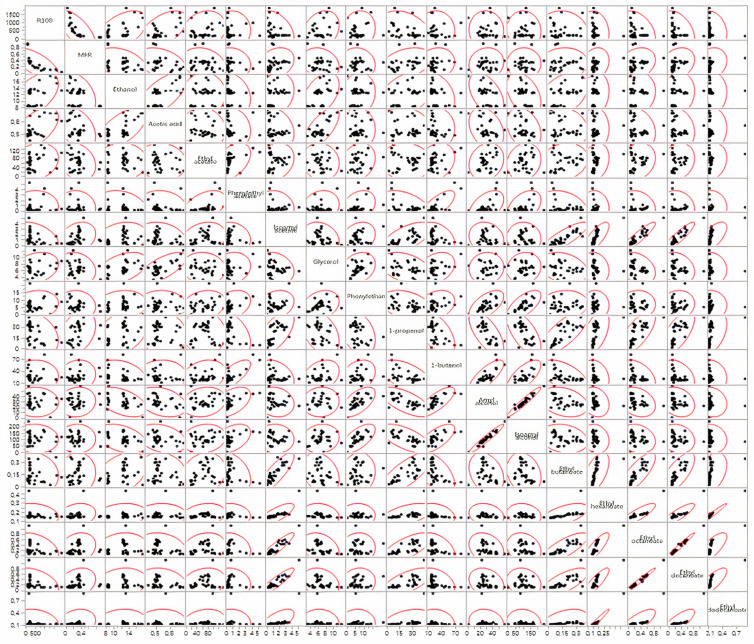
Matrix scatterplot for the 18 responses.

**Figure 3:**
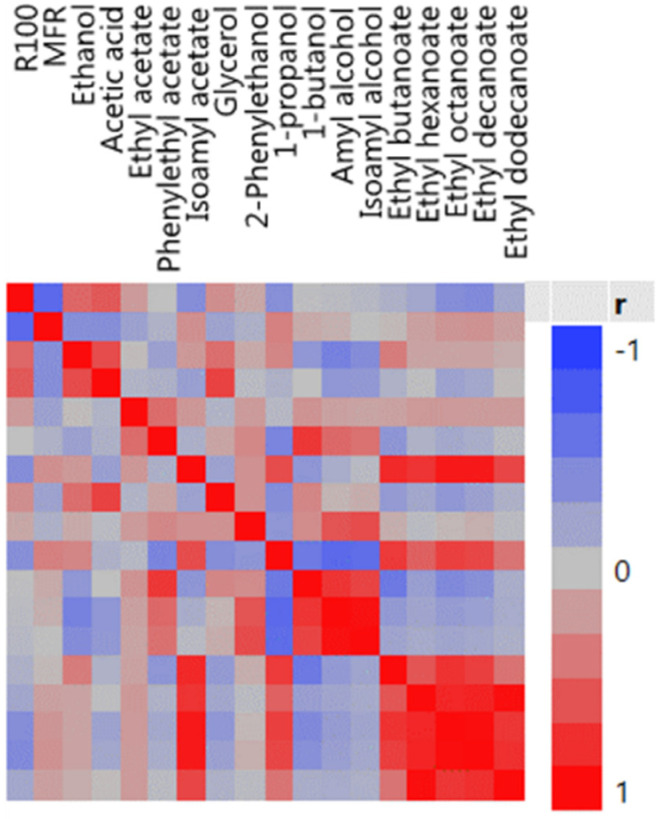
Correlation map of the 18 responses.

In the EDA stage, the pairwise association patterns exhibited by the responses were also analyzed, namely through the matrix of scatterplots (Figure 2) and the color map of correlations (Figure 3). This last plot summarizes, in a simpler way, the association patterns depicted in Figure 2, highlighting those that are more significant; however, a careful analysis of the matrix of scatterplots is recommended to detect possible abnormal observations and analyze the main patterns of association.

Both plots revealed aggregated patterns of correlation, corroborating the existence of modules or blocks of functionally related response variables. This observation justifies the need to incorporate the modular structure of data in the analysis, particularly in the development of the predictive models.

### 3.2. Unsupervised Machine Learning

**Figure 4:**
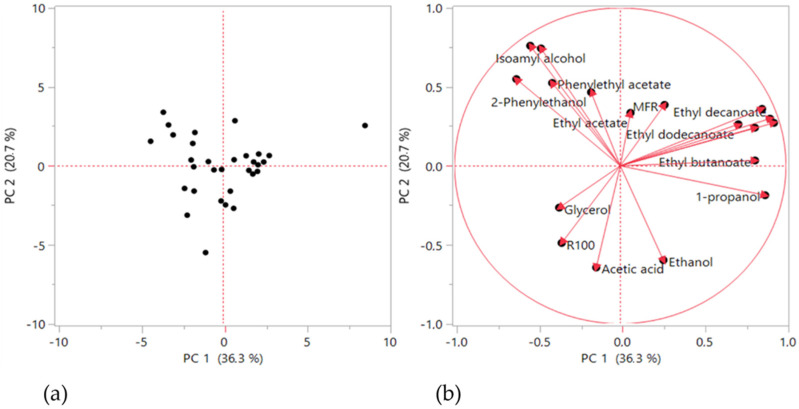
Principal Component Analysis (PCA) analysis of the 18 responses: (**a**) scores plot and (**b**) loadings plot for the first two principal components (PC1 explains 36.3% of the overall variability and PC2 explain 20.7%).

The analysis of the overall correlation structure of the 18 response variables initiated in stage 1 was further complemented using Machine Learning techniques in stage 2, namely PCA. All variables were preliminarily auto scaled (i.e., z-transformed, or transformed to zero mean and unit standard deviation), in order to focus the analysis strictly on the correlation structure. Figure 4 presents the results in the form of a score plot (Figure 4a) and a loading plot (Figure 4b).

**Figure 5:**
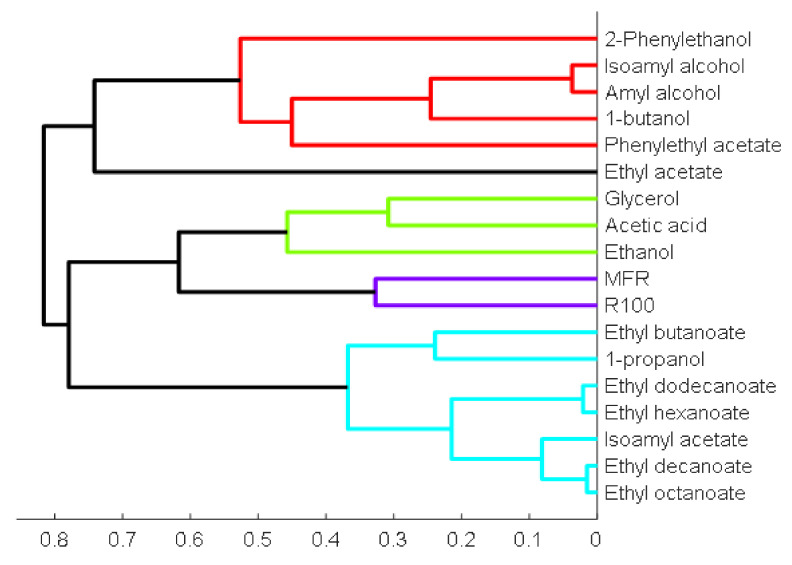
Dendrogram for AHC using a correlation-based distance for detecting modules of associated variables.

The former plot contains information about the distributions of values of the responses, whereas the second one addresses the relationships between the responses. From the score plot, it is possible to observe that the data are nicely scattered in a two-dimensional plane, with observation 31 lying further apart from the rest. This observation was scrutinized in detail and, having found nothing wrong with any of the measurements, it was preserved in the analysis. From the loading plots, again, clear patterns of associated variables (i.e., those falling close together or lying in opposite directions, indicating positive and negative correlations, respectively) can be observed. This preliminary PCA analysis confirms the need to further explore the modular structure of the responses and extract the natural blocks of associated responses. In fact, responses related to the same fundamental phenomenon tend to present higher levels of mutual association. Therefore, another unsupervised machine methodology was applied, namely an agglomerative hierarchical variable-clustering approach (AHC), in order to unravel the natural blocks or clusters of variables. This methodology was implemented using a correlation-based distance measure for identifying the natural modules (clusters). The clustering results are presented in Figure 5.

The groups identified by applying AHC are shown in the dendrogram presented in Figure 5 with different colors (see also the first two columns of Table 3 for the composition of each cluster). In this plot, the shorter the agglomeration distances (i.e., the branches connecting the variables), the more similar they are. In one case, one cluster is formed by a single variable, which exhibits a rather unique behavior (ethyl acetate). In the next stage of the workflow, the clusters identified were analyzed individually and models were developed for each of them to infer the respective critical experimental factors.

### 3.3. Supervised Machine Learning

In the third stage of the data analysis workflow, the clusters of variables identified were first analyzed using PCA models, and the first component was retained to represent the collective variation of the cluster variables. Due to the PCA properties, PC1 is indeed the linear combination of variables showing the highest explanatory power, and therefore is the best candidate for this purpose, within the family of linear combinations. This cluster-specific PCA analysis is summarized in Table 3, where the PC1 loadings (3rd column; the loading coefficients are useful for interpreting trends in the PC1 scores as well as the model derived from it) are indicated, along with the amount of original variation that each PC is able to explain (4th column; we are particularly interested on the amount of variation explained by PC1, which is given by the first value, shown in bold).

Predictive models were derived for explaining the PC1 score for each cluster, using forward stepwise variable selection for multiple linear regression. This approach is adequate, as predictors (experimental factors and their higher-order terms) are not correlated due to the experimental design followed. The results for the cluster-specific models are presented in columns 5 to 9 of Table 3. More specifically: column 5 presents the coefficient of determination of the estimated model (R^2^); column 6, the terms that were selected to incorporate the model by the forward stepwise variable selection strategy; column 7, the associated beta coefficients (i.e., the coefficients for the auto-scaled variables); column 8, the standard error for the estimated beta coefficients; and finally, column 9 presents the *p*-values for the significance test to each coefficient (the lower the *p*-values, the more significant the associated coefficient is).

It is noteworthy that, while each model is predicting the PC1 score, it is also predicting the original values of the responses as well, as they are easily obtained from the predictors’ scores through the outer product with the PC1 loadings (shown in the 3rd column of Table 3).

The results of the modeling using main effects, 2nd-order interactions, and quadratic terms depicted in Table 3 confirm the wide variations observed in the exploratory analysis section (Figure 1), emphasizing the significant impact of the factors on the fermentation process. Some models present very good prediction accuracies (R^2^ > 0.9), while others show lower explanation power (0.53–0.64). The lower R^2^ could be due to experimental variation inherent to the biological nature of yeast fermentations or to analytical random sources of variability that are intrinsic to volatile aroma compounds analysis, such as their volatility, or to the interference of other factor(s) that were not accounted in our experimental model in the production of these compounds. Nevertheless, these lower R^2^ values do not pose any fundamental obstacle in the present study, which is focused on understanding how the four experimental factors affect the responses, for which the statistical significance of the effects in the model, rather than the R^2^, is the most relevant analysis outcome. In the remainder of this section, we comment on the effects of the experimental factors on families of compounds, based on the results of the cluster-specific models. The main factors driving the variability of most clusters can be identified (all models were statistically significant), and clusters 1, 2 and 4 were well predicted.

Overall, analyzing the models derived for all clusters, it is possible to observe that YAN concentration was the factor with the greatest effect on many yeast-derived compounds and/or on fermentation parameters. Its main (or linear) effect was always positive (clusters #2 and #3), except for higher alcohols, 2-phenylethanol and its corresponding acetate ester (cluster #5). This result corroborates the recognized pivotal role of nitrogen on governing yeast growth and fermentative activity [15,50], as well as on the modulation of volatile compounds released in wine [51,52,53]. The effect of fermentation temperature was positive both on fermentation rate and on the production of 2-phenylethanol and 2-phenylethyl acetate (clusters #2 and #5). Sugar content and the inoculum level of *H. guilliermondii* showed moderate to significant effects, either positive or negative, depending on the cluster under analysis.

The model resulted in a single cluster for ethyl acetate, cluster #1, the concentration of which was most significantly modulated by the inoculum level (*p*-value = 2.81 × 10^−7^), with results in line with several other studies that indicate that non-*Saccharomyces* yeast strains are proficient in ester production [7,17,23,25,54]. Additionally, it was possible to verify a negative interaction between inoculum level and initial YAN content in grape must, underlining that the presence of *H. guilliermondii* promotes an increase in ethyl acetate, particularly at lower YAN concentrations (Table S1), suggesting that the overproduction of ethyl acetate in co-fermentations with *H. guilliermondii* can be prevented by the modulation of the initial nitrogen content. Fermentation parameters MFR and R100, included in cluster #2, were significantly impacted by linear effects of all factors, as well as by the interaction of YAN and temperature and quadratic effects of sugar and YAN (Table 3). In line with the results depicted in Figure 1, the rate and duration of fermentation were significantly affected by temperature (*p*-value = 1.05 × 10^−13^), followed by the initial sugar concentration (*p*-value = 2.21 × 10^−11^), initial YAN concentration (*p*-value = 4.72 × 10^−9^) and inoculum level (*p*-value = 0.0139). Accordingly, the fermentation length was longer in the trials conducted at lower temperature (10 °C). Additionally, the negative effect of initial sugar levels on fermentation, either in MFR or in R100, was demonstrated in the fermentation profiles (Figure S1), where the highest initial concentrations of sugar led to longer fermentations, where yeast cells were unable to consume all sugars. The negative quadratic effect of YAN on fermentation length is in line with previous observations by our and other research groups reporting an inverse correlation between nitrogen concentration and the fermentation length, maximum fermentation rate, and final biomass [15,50,55,56,57,58]. Regarding maximal fermentation rate, it was positively affected by initial YAN content, with the highest values being observed for fermentations conducted at high temperature and high YAN concentrations, with the lowest levels of sugar (Table 1, Figure S1D). Indeed, it has been reported that yeast nitrogen requirements are reliant on the amount of sugars present; the higher the initial sugar concentration, the more nitrogen will be needed to complete sugar fermentation [59]. On the other hand, higher fermentation rates have been associated with high nitrogen conditions due to the higher final yeast cell biomass [15], which is in line with our results (Figures S1 and S2). Additionally, it should be mentioned that co-inoculation with *H. guilliermondii* presented a negative effect, which was highly dependent on nitrogen levels and extremes of temperature; this is in line with the improved ability of *Hanseniaspora* spp. To survive and persist in fermentation at lower temperatures, as their susceptibility to ethanol is attenuated under such conditions [35]. Accordingly, yeast growth profiles (Figure S2) show that *H. guilliermondii* UTAD222 sustained a higher number of viable cells during fermentations at lower temperatures. On the contrary, runs 16 and 17, corresponding to extreme conditions (150 g/L of sugar, 500 mg/L YAN, 30 °C; Figure S1D), resulted in short fermentation periods (96 h), in which the presence of *H. guilliermondii* had no effect. Interestingly, in these experiments, the effectiveness of temperature in decreasing *H. guilliermondii* cell viability was not clear (Figure S2D), as high cell concentrations were sustained. This observation is probably due to the high tolerance to ethanol of the *H. guilliermondii* strain used in this study, up to 9% [17]. Moreover, its presence slightly improved fermentation efficiency, as can be seen for run 17, probably due to the high carbon/nitrogen ratio in the medium, which prevented the potential competition for nutrients by both strains.

As shown in Figure S1, for central point conditions, all fermentations were finished after 216 h, whereby the presence of *H. guilliermondii* did not interfere with the fermentation length, irrespective of the inoculum level (Figure S1C). This observation is somehow surprising, since previous experiments conducted under similar temperature conditions, with nitrogen conditions within the range used in this study, and with the same yeast strains [17] showed a negative effect of *H. guilliermondii* co-inoculation on the fermentative activity of *S. cerevisiae* UCD522. This conflicting result could be due to differences in culture media composition, since in this work a synthetic grape juice medium was used and in the previous study the fermentations were conducted with a natural grape juice.

Ethyl esters and isoamyl acetate, along with 1-propanol, included in cluster #3, were shown to be significantly dependent on YAN (*p*-value = 0.0052) and on the quadratic effect of initial sugar levels (*p*-value = 4.58 × 10^−6^). Contrary to the well-established inverse relationship between nitrogen concentration and production of alcohols, propanol levels were positively affected by nitrogen (Table S1), in line with results obtained by others [53,60]. In particular, Mouret et al. [60] identified this compound to be both a metabolic marker of intracellular nitrogen availability and a quantitative marker of assimilable nitrogen levels.

Additionally, the production of isoamyl acetate and several ethyl esters mirrored the recognized positive association with the initial nitrogen content [17,50,61]. Surprisingly, no effect of the inoculum level resulted from the model, which is in contrast to the recognized role of non-*Saccharomyces* wine yeasts as good producers of esters (reviewed in [25]). Indeed, Barbosa et al. [23], using a transcriptomic-based approach, showed that the expression of genes of *S. cerevisiae* involved in the biosynthesis of these compounds is higher when in co-culture with *H. guilliermondii*, suggesting that the presence of this non-*Saccharomyces* strain contributes to greater production of ethyl esters.

Analyzing the model for cluster #4, it is possible to verify that the production of the primary products of alcoholic fermentation, ethanol, glycerol and acetic acid, which have an important contribution to the aroma perception of wine [62], showed a significantly high positive effect of sugar, either in single (*p*-value = 1.32 × 10^−16^) or quadratic term (*p*-value = 2.71 × 10^−4^), and negative effects of the interaction of the nitrogen concentration with temperature (*p*-value = 5.76 × 10^−4^) and of inoculum level (*p*-value = 0.0032).

As previously mentioned, most fermentations conducted with 300 g/L of sugars stopped prematurely (Table 2), leaving residual sugars in the medium and attaining final ethanol concentrations ranging from 12.5 to 17.7% (*v*/*v*). On the other hand, the final concentrations of ethanol varied from 8.4 to 8.7% and from 12.4 to 13.5% (*v*/*v*) in the fermentations conducted with 150 or 225 g/L of initial sugar, respectively. Additionally, the highest ethanol levels were obtained using the highest nitrogen levels and the lowest temperature, in line with results obtained in runs 7 and 27 (300 g/L of sugars, 500 mg/L YAN, 10 °C). These observations might explain the significant quadratic effect of initial sugar levels (*p*-value = 2.71 × 10^−4^) and the interaction of nitrogen level and temperature (*p*-value = 5.76 × 10^−4^) on ethanol production. The negative effect of co-inoculation with *H. guilliermondii* (Table 3) should also be highlighted: a decrease in ethanol produced was noticed when the medium was co-inoculated (run 7—17.2%) compared with *S. cerevisiae* in single culture (run 27—17.7%) (Table S1); this result is in agreement with previous observations of ethanol reduction in wines produced from mixed-culture fermentations with non-*Saccharomyces* and with *S. cerevisiae* (reviewed in [40]).

Additionally, glycerol and acetic acid concentrations were found to be directly related to sugar concentration; the higher the sugar concentration, the greater the amount of acetic acid and glycerol produced. The direct relationship between acetic acid formation and sugar concentration was clearly demonstrated by Erasmus et al. [63] with a range of sugar amounts of 20–50% using seven commercial wine yeast strains. For all combinations tested, the maximum levels of glycerol were achieved in run 29 (300 g/L of sugars, 100 mg/L YAN, 30 °C), whereas the lowest were obtained in run 17 (150 g/L of sugars, 500 mg/L YAN, 30 °C). The higher glycerol production observed in fermentations conducted with higher sugar content was expected, because its major involvement in yeast cell redox homeostasis during fermentation, as well as in the metabolic stress response to osmolarity, is well known [64].

On the contrary, the production of these metabolites was negatively affected by the interaction of temperature and YAN available, and by co-inoculation with *H. guilliermondii* (Table 3). Concentrations of glycerol and acetic acid were significantly reduced at lower temperatures, but the magnitude of this effect was found to depend on the amount of YAN present, as demonstrated by the negative effect of the interaction of YAN and temperature (Table 3). As shown in Table S1, the lowest glycerol concentration was produced at high YAN content (run 17), while the lowest acetic acid level was attained at moderate YAN concentrations (run 22), probably due to the different fermentation temperatures, 30 and 20 °C, respectively. Our results suggest a positive interaction between YAN concentration and the temperature variation on the amount of yeast primary products of alcoholic fermentation, in agreement with those reported by Mouret et al. [60]. Interestingly, the inoculum of *H. guilliermondii* was found to contribute to control/reduce acetic acid production by *S. cerevisiae* in mixed-culture fermentations (Table S1), contrary to what has been observed in other studies [7,65,66,67]. Even though a desirable increase of acetate esters in mixed fermentations of Macabeo and synthetic must with *H. uvarum*/*S. cerevisiae* was reported, the high concentration of acetic acid hampered the industrial application of the mixed starter [66]. Accordingly, in the fermentations conducted with 150 g/L of sugar, the amount of acetic acid produced ranged from 0.34 to 0.47 g/L in mixed-culture or from 0.50 to 0.62 g/L in single-culture *S. cerevisiae* fermentations. Similarly, reports with other non-*Saccharomyces* species, namely *Kluyveromyces lactis* [68], *Torulaspora delbrueckii* [69] and *Candida zemplinina* [70], have also shown the same potential for reducing acetic acid levels in co-inoculation regimes with *S. cerevisiae* strains. Production of glycerol according to our model is, in contrast to previous results [23], significantly influenced by co-inoculation with *H. guilliermondii* (*p*-value = 0.0032) and modulated by the interaction of YAN levels and temperature of fermentation (*p*-value = 5.76 ×10^−4^). The effect of non-*Saccharomyces* yeasts on the reduction of ethanol and glycerol contents in wine, extensively reported, was analyzed in a recent review with respect to inoculum timing and rate, as well as aeration conditions [40].

Regarding the model for cluster #5, it is possible to observe that the production of higher alcohols and 2-phenylethyl acetate were significantly impacted by linear effects of YAN and temperature, as well as by the interaction of sugar and inoculum (Table 3). These compounds with positive impact on the aroma of wine, showed a significantly high negative effect of YAN (*p*-value = 1.99 × 10^−4^), proved by the higher production in media with low YAN content (Table S1) and in line with previous studies that pointed nitrogen availability as the main fermentation parameter affecting the kinetics of higher alcohol synthesis [50,53,60]. Moreover, the model for this cluster underlined the importance of fermentation temperature in the modulation of the synthesis of these volatile compounds, as shown by the positive linear effect of the temperature (*p*-value = 0.0095), which translated into high contents of higher alcohols and 2-phenylethyl acetate in the experiments conducted at the highest temperature (Table S1). In particular, the wines obtained in runs 9 and 24 were those with the highest content of 2-phenylethyl acetate and most of the higher alcohols. The positive effect of temperature detected herein has already been studied and demonstrated by others using commercial *S. cerevisiae* strains [53,60], or using two non-*Saccharomyces* yeasts, *H. uvarum* and *C. membranefaciens*, with the sequential inoculation with *S. cerevisiae* on Malbec must [4].

It is noteworthy that the production of higher alcohols and 2-phenylethyl acetate differed according to sugar availability, and it was dependent on the inoculum level (Table 3 and Table S1), as demonstrated by the positive effect of the interaction between sugar availability and co-inoculation with *H. guilliermondii* (*p*-value = 0.0450). The increase of fruity acetate esters, in particular 2-phenylethyl acetate, has been the main target of mixed starters designed with *Hanseniaspora* species, as also reported by others when using *H. guilliermondii* and *H. vineae* strains in mixed culture with *S. cerevisiae* [17,54,71]. In our study, the positive effect of the interaction between sugar and inoculum level was clearly seen in the highest content of 2-phenylethyl acetate detected in run 24 (Table S1), in which sugar and inoculum of *H. guilliermondii* were at the highest loads.

## 4. Conclusions

The analysis conducted in this study is the first of its kind to offer a comprehensive analysis of *S. cerevisiae* fermentation kinetics and chemical composition landscape under different environmentally relevant fermentation conditions in terms of nitrogen and sugar contents, temperature, and inoculum level of non-*Saccharomyces* yeast strains. The main purpose was to systematically determine the relevant factors and interactions between the studied winemaking parameters and fermentation chemical compounds. Even though the optimization of metabolite production was not established as a research goal, the polynomial regression models developed enabled the estimation of optimal values, but the resulting data were not subjected to confirmatory fermentation analysis. With the data analysis workflow designed for this study, we were able to clearly identify and handle the clustering structure of results and summarizing it in an efficient way using cluster specific PCA models. The main factors driving the variability of the clusters were identified, particularly for the well predicted clusters 1, 2 and 4, demonstrating that the presence of *H. guilliermondii* UTAD222, even though presenting a negative impact on fermentation rate and length, contributed to the increase of ethyl acetate and to the decrease in acetic acid and ethanol levels. The results generated using supervised and unsupervised machine learning techniques also suggest that it is possible to increase the aromatic and fermentative potential of *H. guilliermondii* UTAD222 by modulating temperature and the availability of nitrogen and/or sugars in the medium, which would directly affect the final concentrations of certain volatile compounds. Overall, this study gathered knowledge to guide the rational development of mixed blends that can be used to a specific wine style as a function of fermentation conditions.

## Figures and Tables

**Table 1:** Factor ranges and levels considered in the experimental design (CCD), expressed both in terms of coded and original values.

Level	Sugar (g/L)	YAN (mg/L)	Temperature (°C)	*H. guilliermondii* (CFU/mL)
−1	150	100	10	0
0	225	300	20	5 × 10^5^
1	300	500	30	1 × 10^6^

**Table 2:** Experimental conditions used following the CCD experimental plan. Shaded lines represent the seven replicates at the center of the experimental domain.

Run	Sugars (g/L)	YAN (mg/L)	Temperature (°C)	*H. guilliermondii* (CFU/mL)
1	225	300	20	5 × 10^5^
2	150	100	30	0
3	150	500	10	0
4	150	100	10	0
5	225	300	20	5 × 10^5^
6	225	300	30	5 × 10^5^
7	300	500	10	1 × 10^6^
8	300	100	10	1 × 10^6^
9	150	100	30	1 × 10^6^
10	300	500	30	0
11	300	300	20	5 × 10^5^
12	225	300	20	0
13	225	300	10	5 × 10^5^
14	225	300	20	5 × 10^5^
15	225	300	20	1 × 10^6^
16	150	500	30	0
17	150	500	30	1 × 10^6^
18	150	500	10	1 × 10^6^
19	225	300	20	5 × 10^5^
20	225	300	20	5 × 10^5^
21	300	500	30	1 × 10^6^
22	150	300	20	5 × 10^5^
23	300	100	10	0
24	300	100	30	1 × 10^6^
25	225	100	20	5 × 10^5^
26	150	100	10	1 × 10^6^
27	300	500	10	0
28	225	300	20	5 × 10^5^
29	300	100	30	0
30	225	300	20	5 × 10^5^
31	225	500	20	5 × 10^5^

**Table 3:** Summary of results: modeling using main effects, 2nd-order interactions and quadratic terms.

Cluster Id	Cluster Composition	Loadings PC1	PCA—CumSum of Explain Variance	Model R^2^	Selected Regressors	Beta Coefficient	Std. Err	*p*-Value
1	Ethyl acetate	1	100.0000	0.6409	I	0.7592	0.1132	2.81 × 10^−7^
					N × I	−0.2088	0.0930	0.0329
2	R100	−0.7071	83.6559	0.9588	S	−0.6569	0.0547	2.21 × 10^−11^
	MFR	0.7071	100.0000		N	0.4962	0.0547	4.72 × 10^−9^
					T	0.8519	0.0547	1.05 × 10^−13^
					I	−0.1458	0.0547	0.0139
					N × T	0.1663	0.0450	0.0012
					S^2^	−0.2045	0.0966	0.0452
					N^2^	−0.2727	0. 0966	0.0096
3	Ethyl hexanoate	0.3857	78.5281	0.5956	N	0.8544	0.2818	0.0052
	Ethyl dodecanoate	0.3494	91.6550		S^2^	−1.9078	0.3371	4.58 × 10^−6^
	Isoamyl acetate	0.4000	96.2827					
	Ethyl octanoate	0.4157	99.0158					
	Ethyl decanoate	0.4122	99.8665					
	1-propanol	0.3231	99.9728					
	Ethyl butanoate	0.3492	100.0000					
4	Ethanol	0.5446	73.0421	0.9380	S	1.3519	0.0723	1.32× 10^−16^
	Acetic acid	0.6202	91.5613		I	−0.2347	0.0723	0.0032
	Glycerol	0.5646	100.000		N × T	−0.2327	0.0594	5.76 × 10^−4^
					S^2^	0.3637	0.0864	2.71 × 10^−4^
5	2-Phenylethanol	0.3658	69.2298	0.5323	N	−1.0532	0.2449	1.99 × 10^−4^
	Phenylethyl acetate	0.3737	86.7186		T	0.6838	0.2449	0.0095
	1-butanol	0.4725	96.9133		S × I	0.4231	0.2012	0.0450
	Amyl alcohol	0.5063	99.4044					
	Isoamyl alcohol	0.4969	100.0000					

S—initial sugar content; N—initial YAN concentration; T—temperature of fermentation; I—inoculum level of the non-*Saccharomyces* strain *H. guilliermondii* UTAD222.

## Supplementary Materials

The following are available online at https://www.mdpi.com/article/10.3390/microorganisms10010107/s1, Figure S1: Fermentation profiles in selected experimental conditions: (**A**) 150 g/L of sugar, 100 mg/L YAN, 10 °C; (**B**) 300 g/L of sugar, 100 mg/L YAN, 10 °C; (**C**) 225 g/L of sugar, 300 mg/L YAN, 20 °C (Center conditions); (**D**) 150 g/L of sugar, 500 mg/L YAN, 30 °C; (**E**)—300 g/L of sugar, 500 mg/L YAN, 30 °C, inoculated with *S. cerevisiae* UCD522 in single (red) or in mixed-culture with *H. guilliermondii* UTAD222 (green), Figure S2: Yeast growth profiles in selected experimental conditions, inoculated with *S. cerevisiae* UCD522 in single (red) or in mixed-culture with *H. guilliermondii* UTAD222 (green). (**A**) 150 g/L of sugar, 100 mg/L YAN, 10 °C; (**B**) 300 g/L of sugar, 100 mg/L YAN, 10 °C; (**C**) 225 g/L of sugar, 300 mg/L YAN, 20 °C (Center conditions); (**D**) 150 g/L of sugar, 500 mg/L YAN, 30 °C; (**E**) 300 g/L of sugar, 500 mg/L YAN, 30 °C. Table S1: Values of fermentation parameters and concentrations of metabolites produced, under the thirty-one experiments.
