# Supplementary material for: Machine Learning Techniques Disclose the Combined Effect of Fermentation Conditions on Yeast Mixed-Culture Dynamics and Wine Quality
Source: Microorganisms. 2022 Jan 5;10(1):107. doi: 10.3390/microorganisms10010107 (PMC8781278; doi:10.3390/microorganisms10010107)
Supplement: Supplementary file 1 [file microorganisms-10-00107-s001.zip › microorganisms-1508185-supplementary-update/microorganisms-1508185-supplementary.docx]

Figure S1: Fermentation profiles in selected experimental conditions: (**A**) 150 g/L of sugar, 100 mg/L YAN, 10 °C; (**B**) 300 g/L of sugar, 100 mg/L YAN, 10 °C; (**C**) 225 g/L of sugar, 300 mg/L YAN, 20 °C (Center conditions); (**D**) 150 g/L of sugar, 500 mg/L YAN, 30 °C; (**E**)—300 g/L of sugar, 500 mg/L YAN, 30 °C, inoculated with *S. cerevisiae* UCD522 in single (red) or in mixed-culture with *H. guilliermondii* UTAD222 (green),


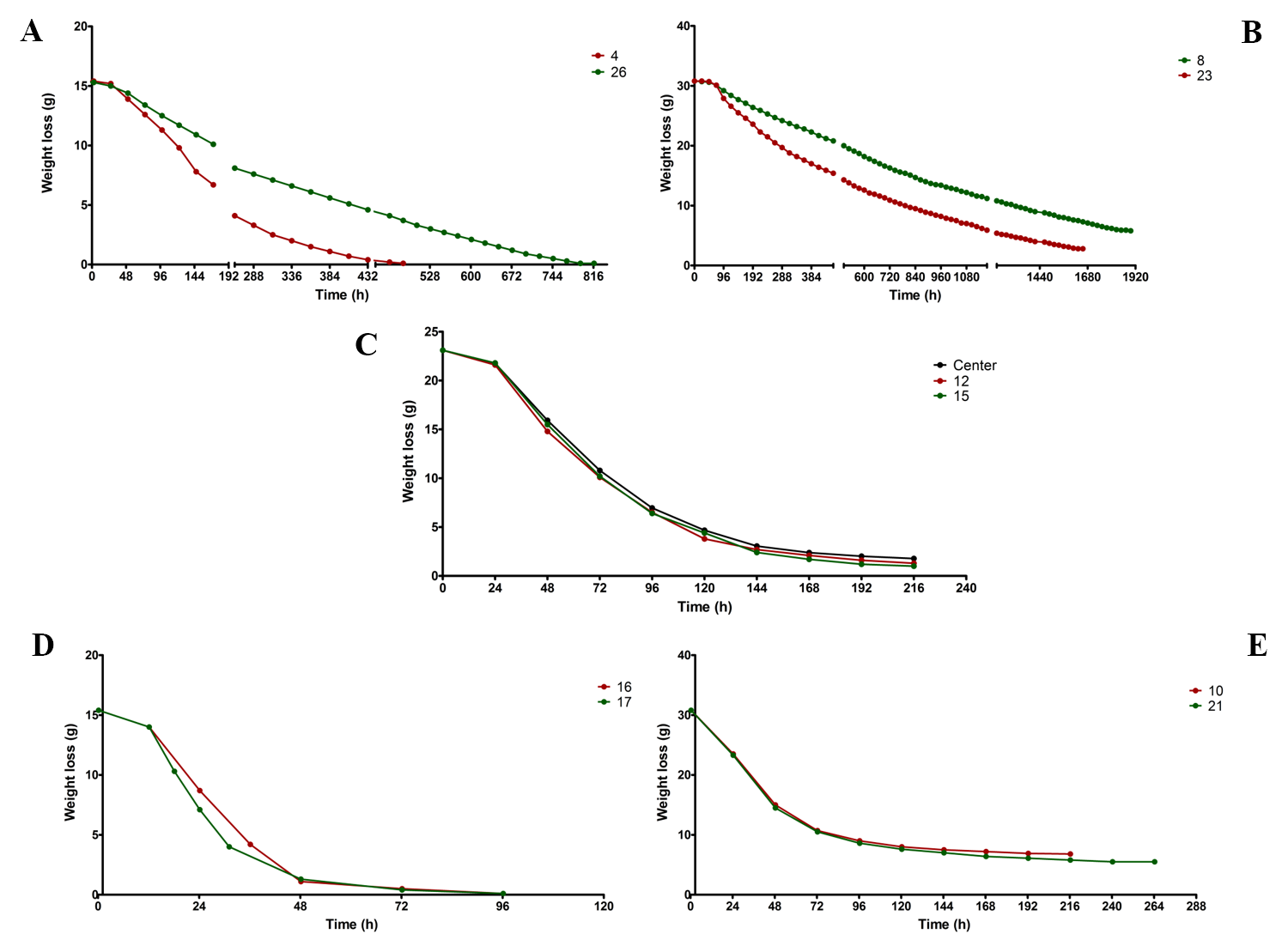


Figure S2: Yeast growth profiles in selected experimental conditions, inoculated with *S. cerevisiae* UCD522 in single (red) or in mixed-culture with *H. guilliermondii* UTAD222 (green). (**A**) 150 g/L of sugar, 100 mg/L YAN, 10 °C; (**B**) 300 g/L of sugar, 100 mg/L YAN, 10 °C; (**C**) 225 g/L of sugar, 300 mg/L YAN, 20 °C (Center conditions); (**D**) 150 g/L of sugar, 500 mg/L YAN, 30 °C; (**E**) 300 g/L of sugar, 500 mg/L YAN, 30 °C.


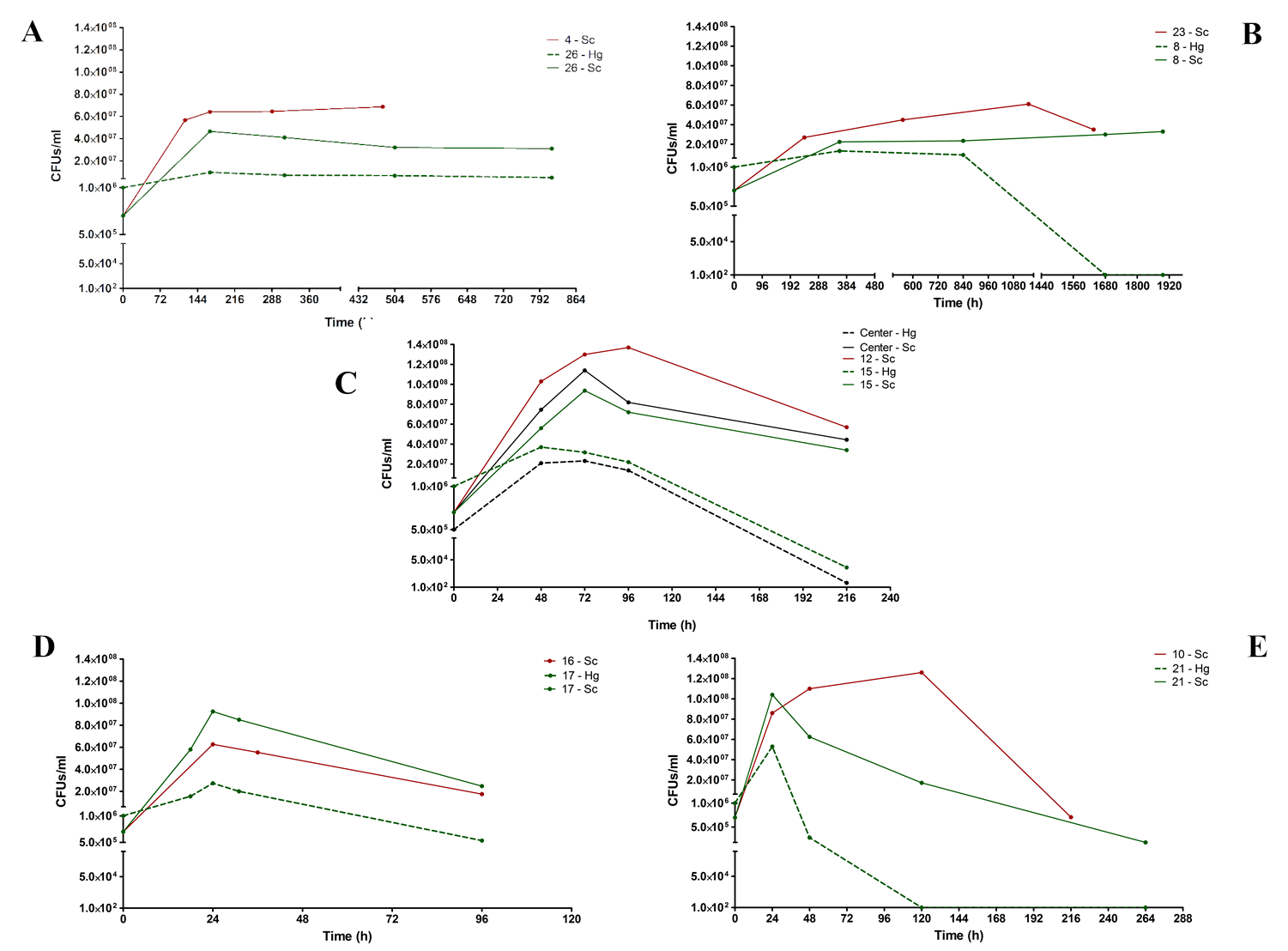


Table S1: Values of fermentation parameters and concentrations of metabolites produced, under the thirty-one experiments.
