# Supplementary figures and images for: Machine Learning Techniques Disclose the Combined Effect of Fermentation Conditions on Yeast Mixed-Culture Dynamics and Wine Quality
Source: Microorganisms. 2022 Jan 5;10(1):107. doi: 10.3390/microorganisms10010107 (PMC8781278; doi:10.3390/microorganisms10010107)

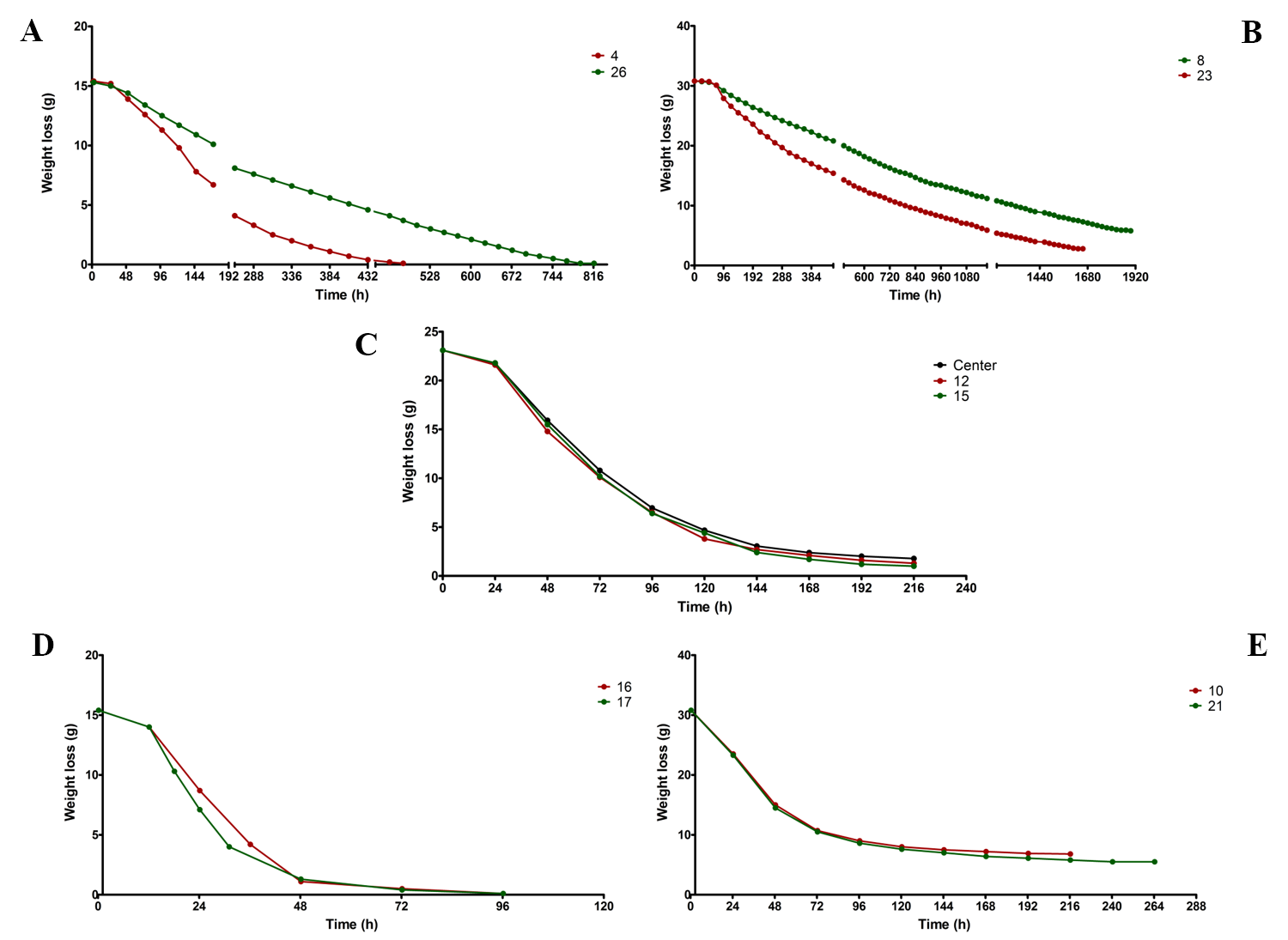

Supplement: Supplementary file 1 [file microorganisms-10-00107-s001.zip › microorganisms-1508185-supplementary-update/Figure S1.tif]

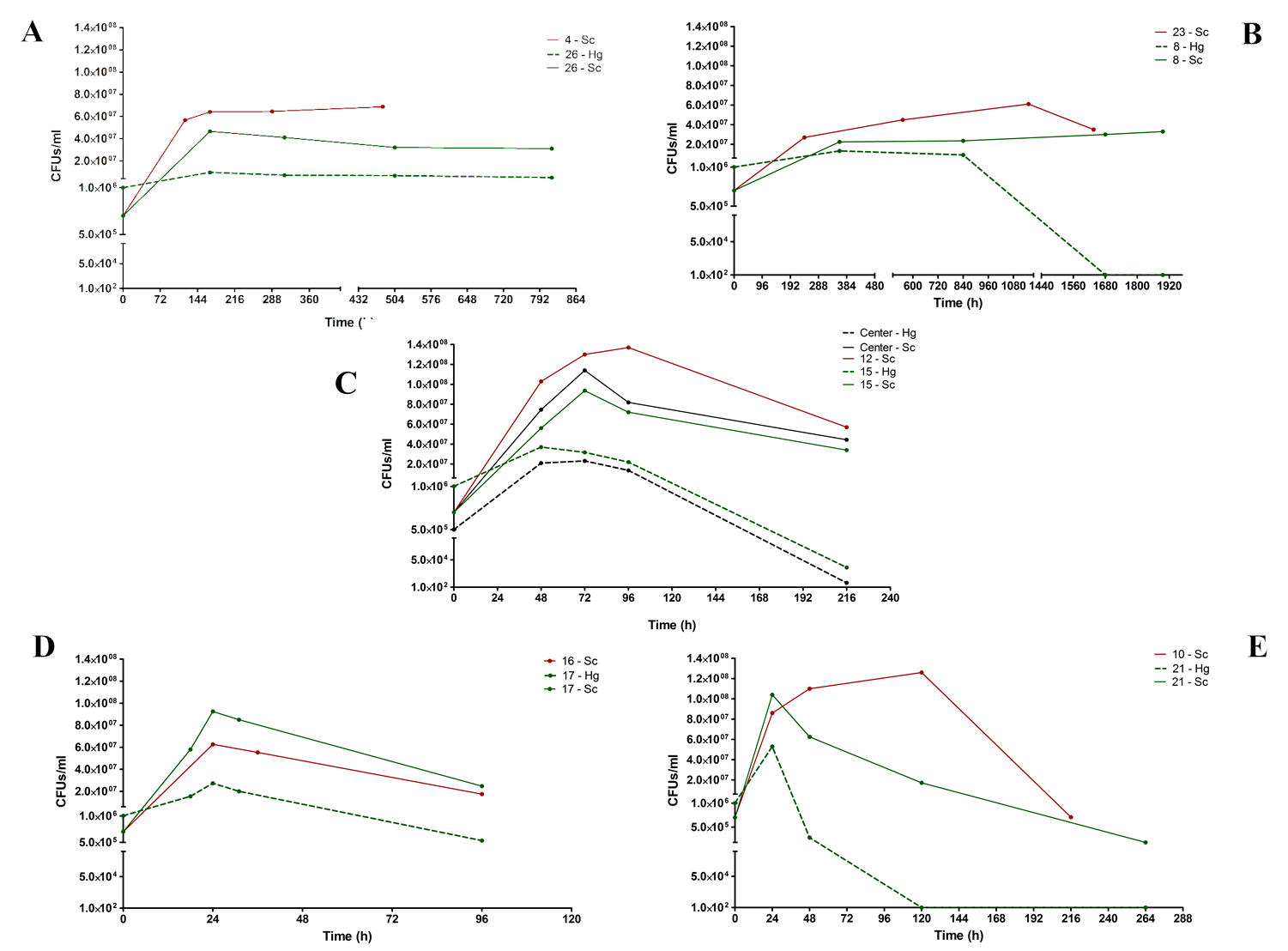

Supplement: Supplementary file 1 [file microorganisms-10-00107-s001.zip › microorganisms-1508185-supplementary-update/Figure S2.tif]
